# Increasing Cardiac Myosin Super‐Relaxation With Decreasing Metabolic Demand

**DOI:** 10.1161/JAHA.124.035479

**Published:** 2024-06-11

**Authors:** Julien Ochala, Carlos Galán‐Arriola, Vebjørn Veiberg, Borja Ibanez

**Affiliations:** ^1^ Department of Biomedical Sciences University of Copenhagen Copenhagen Denmark; ^2^ Myocardial Homeostasis and Cardiac Injury Program Centro Nacional de Investigaciones Cardiovasculares (CNIC) Madrid Spain; ^3^ CIBER de Enfermedades Cardiovasculares (CIBERCV) Madrid Spain; ^4^ Department of Terrestrial Ecology Norwegian Institute for Nature Research Trondheim Norway; ^5^ Cardiology Department IIS‐Fundación Jiménez Díaz University Hospital Madrid Spain

**Keywords:** heart, hibernating myocardium, metabolism, minipig, myosin, reindeer, Metabolism, Myocardial Biology, Chronic Ischemic Heart Disease, Heart Failure

Ischemic heart disease often leads to heart failure with reduced ejection fraction and to a puzzling condition termed hibernating myocardium, where the left ventricle is dysfunctional but still viable.^1^ Strikingly, the hibernating myocardium experiences a change in its metabolism and a remodeling of its energy supply.^2^ This undoubtedly underlies the involvement of mechanisms generating ATP. Six kilograms of ATP are hydrolyzed every day by the human heart. The major cardiac energy‐demanding cycles include the sarcoendoplasmic reticulum Ca^2+^ ATPases (to pump free Ca^2+^ back into the sarcoplasmic reticulum) and myosin ATPases. Hence, in the present study, we aimed to verify the hypothesis that, as a consequence of hypoperfusion, the hibernating myocardium has myosin‐related ATP‐consuming cycles being partially shut down via accrued myosin biochemical super‐relaxation. For that, following Animal Research Committee approvals, left ventricle samples were obtained from control Yucatan minipigs (N=7) and animals undergoing a surgical casein ameroid implant around the proximal left anterior descending coronary artery, inducing a reduced ejection fraction (<50%) and hibernating myocardium (N=7).^2^ Samples from animals with a previously described phenotype^2^ were then dissected into thin bundles and membrane‐permeabilized. Thin cardiac strips (≈50‐μm wide) were isolated and we applied a loaded Mant‐ATP chase assay (7–8 strips per animal per condition).^3^ In line with a previous study in patients with heart failure,^3^ at short sarcomere length of 2.0 μm, we observed a significantly higher proportion of super‐relaxed (SRX) myosins in the hibernating myocardia than in controls (Figure A). When cardiac strips were stretched to a sarcomere length of 2.3 μm, mimicking diastolic events, we found no difference in the percentage of SRX myosins between conditions (Figure B). Interestingly, when we exposed cardiac strips to 1 μmol/L Mavacamten, known to be a potent myosin ATPase inhibitor and activator of myosin super‐relaxation, the difference was abolished (Figure C). Similarly, when cardiac strips were incubated with 10 μmol/L EMD57033, known to be a motor activator and myosin super‐relaxation inhibitor, no difference was seen (Figure D). Taken together, as initially hypothesized, our data confirm that myosin‐related ATP‐consuming cycles are partially shut down in the hibernating myocardium by enhancing the number of cardiac myosins blocked in the ATP‐conserving SRX. To further determine whether the results relate to myosin structural auto‐inhibited state (=electrostatic interactions between myosin head and coiled‐coil region), we repeated the loaded Mant‐ATP chase experiments at various ionic strengths by gradually increasing the KCl concentration from 25 to 150 mmol/L. In controls, the proportion of SRX myosins decreased with increasing ionic strength whereas in hibernating myocardia, the ionic strength dependence was less pronounced (Figure E). These results then strongly indicate that the promotion of myosin super‐relaxation in the hibernating myocardium is paired with an accrued structural myosin auto‐inhibited/stabilized state. The data that support the findings of this study are available from the corresponding author upon reasonable request.

**Figure .**
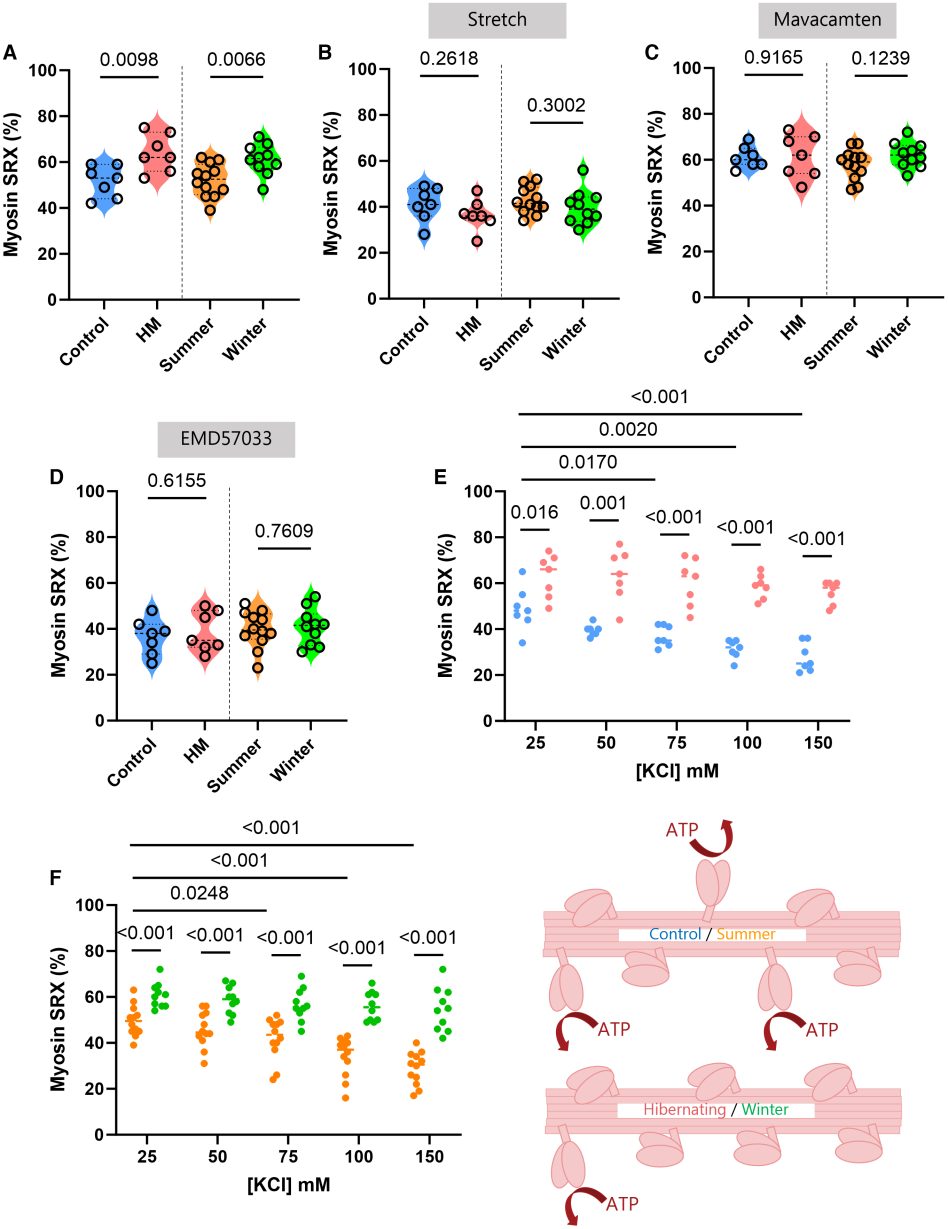
Regulation of myosin super‐relaxation when the energetic demand is depressed. Thin left ventricle strips were isolated from Yucatan minipigs (N=7 controls and N=7 animals with hibernating myocardium [HM]) and from Svalbard reindeer (N=12 for summer and N=10 for winter). Mant‐ATP chase experiments were carried out to estimate the percentage of SRX myosins under various conditions. Briefly, cardiac strips were individually mounted in a home chamber. Seven to 8 strips were tested per animal per condition. Data were fit to an unconstrained double exponential decay. Here we present the amount of SRX myosins at a sarcomere length of 2.0 μm (**A**); at a sarcomere length of 2.3 μm (**B**); in the presence of 1 μmol/L of Mavacamten (**C**); in the presence of 10 μmol/L of EMD57033 (**D**); and with increasing concentrations of KCl (**E**, **F**). Circles correspond to means for individual animals. For panels (**A**), (**B**), (**C**), and (**D**), violin plots are also presented; because data followed a normal distribution, the unpaired *t* test was applied with *P* <0.05 as level of significance. For panels (**E**) and (**F**), 2‐way ANOVAs with repeated measures were performed with *P* < 0.05 as level of significance (Factor 1: animal condition–Factor 2: KCl concentration). At the bottom right of the figure is a cartoon summarizing the findings: cardiac myosin molecules are blocked in the myosin auto‐inhibited and ATP‐conserving SRX in minipigs and Svalbard reindeer. SRX indicates super‐relaxed.

After observing the myosin adaptations upon a cardiac condition (hypoperfusion), we aimed to explore whether this phenomenon also occurs in physiological/natural conditions where cardiac metabolic conditions are reduced. Rather than relying on laboratory animal models, our experiments originally included nonhibernating Svalbard reindeer (*Rangifer tarandus platyrhynchus*). These animals live in the High Arctic and experience extreme seasonal differences both related to activity, food availability, reproductive investment, as well as overall environmental conditions. Among the multitude of adaptations developed to cope with this seasonal variation (and keep a normal cardiac function), animals lower their heart rate during winter, enabling them to adjust their metabolic phenotype.^4^ For the present study, 22 Svalbard reindeer from Reindalen, Semmeldalen, and Colesdalen in Nordenskiöld Land, Svalbard (77°50′–78°20′ N, 15°00′–17°30′ E) were culled in accordance with Norwegian regulations as part of scientific campaigns early August (summer) 2022 (N=12) and late October (winter) 2022 (N=10).^4^ Samples were acquired from the left ventricle free wall and the methodology used on thin cardiac strips was similar as for the pigs. At short sarcomere length of 2.0 μm, the number of SRX myosins was significantly greater during winter than summer (Figure A). At a longer sarcomere length of 2.3 μm, we did not observe any seasonal difference (Figure B). When applying Mavacamten (Figure C) or EMD57033 (Figure D), the proportions of SRX myosins were similar between summer and winter. When increasing ionic strength, the fraction of SRX myosins decreased during summer but not winter (Figure F).

Altogether, even though the underlying molecular mechanisms may be species/condition‐specific, our results in pigs and Svalbard reindeer support the concept that when the heart metabolic demand changes dramatically (either under pathological or physiological conditions), myosin super‐relaxation is used as a strategy to avoid unnecessary ATP‐consuming cycles. Pharmacological compounds, such as Mavacamten, mimicking this accrued number of SRX myosins, would then constitute an efficient therapeutic strategy not only for patients diagnosed with hypertrophic cardiomyopathy (known to have lower proportions of SRX myosins)^5^ but also for other unrelated heart failure conditions where metabolic demand is aberrantly increased.

## Sources of Funding

This work was generously funded by the Carlsberg Foundation (CF20‐0113 and CF23‐1023) grants to J.O. Cardiac tissue samples were collected from animals culled for scientific purposes by the PRISM‐project funded by the Research Council of Norway (project 315 454). Part of this work was funded by the Spanish Society of Cardiology (grant SEC Translacional 2019 to B.I.). The CNIC is supported by the ISCIII, the MICIU, and the Pro CNIC Foundation and is a Severo Ochoa Center of Excellence (CEX2020‐001041‐S).

## Disclosures

None.
